# Gastrointestinal Applications of Iodine Quantification Using Dual-Energy CT: A Systematic Review

**DOI:** 10.3390/diagnostics10100814

**Published:** 2020-10-13

**Authors:** Jack Junchi Xu, Mikkel Taudorf, Peter Sommer Ulriksen, Michael Patrick Achiam, Timothy Andrew Resch, Michael Bachmann Nielsen, Lars Birger Lönn, Kristoffer Lindskov Hansen

**Affiliations:** 1 Department of Diagnostic Radiology, Copenhagen University Hospital, Rigshospitalet, 2100 Copenhagen, Denmark; mbn@dadlnet.dk (M.B.N.); lonn.lars@gmail.com (L.B.L.); Kristoffer.Lindskov.Hansen.01@regionh.dk (K.L.H.); 2 Department of Surgical Gastroenterology, Copenhagen University Hospital, Rigshospitalet, 2100 Copenhagen, Denmark; mikkel.taudorf@regionh.dk (M.T.); peter.sommer.ulriksen@regionh.dk (P.S.U.); michael.patrick.achiam.01@regionh.dk (M.P.A.); timothyresch@gmail.com (T.A.R.); 3 Department of Vascular Surgery, Copenhagen University Hospital, Rigshospitalet, 2100 Copenhagen, Denmark; 4 Department of Clinical Medicine, University of Copenhagen, 2100 Copenhagen, Denmark

**Keywords:** dual-energy CT, iodine quantification, iodine concentration, gastrointestinal diseases, gastrointestinal tract, tumor differentiation

## Abstract

Dual-energy computed tomography (DECT) can estimate tissue vascularity and perfusion via iodine quantification. The aim of this systematic review was to outline current and emerging clinical applications of iodine quantification within the gastrointestinal tract using DECT. The search was conducted with three databases: EMBASE, Pubmed and The Cochrane Library. This identified 449 studies after duplicate removal. From a total of 570 selected studies, 30 studies were enrolled for the systematic review. The studies were categorized into four main topics: gastric tumors (12 studies), colorectal tumors (8 studies), Crohn’s disease (4 studies) and miscellaneous applications (6 studies). Findings included a significant difference in iodine concentration (IC) measurements in perigastric fat between T1–3 vs. T4 stage gastric cancer, poorly and well differentiated gastric and colorectal cancer, responders vs. non-responders following chemo- or chemoradiotherapy treatment among cancer patients, and a positive correlation between IC and Crohn’s disease activity. In conclusion, iodine quantification with DECT may be used preoperatively in cancer imaging as well as for monitoring treatment response. Future studies are warranted to evaluate the capabilities and limitations of DECT in splanchnic flow.

## 1. Introduction

Multi-detector computed tomography (MDCT) is the first-line imaging modality for various conditions ranging from acute bleedings [1] and acute intestinal ischemia [2] to Crohn’s disease [3] and gastrointestinal cancer [4,5]. While MDCT is based on X-ray emissions of one energy level, dual-energy computed tomography (DECT) acquires datasets at two different energy levels, either through emission or through separation at the detector level, providing new imaging and reconstruction possibilities [6]. Current DECT scanners produce images comparable to conventional single-energy computed tomography (CT) without increased radiation exposure or decreased image quality [7,8].

The benefits of DECT range from musculo-skeletal applications with the visualization of gout [9] and bone marrow edema [10] to neurological applications, improving the differentiation between intracranial hemorrhage and contrast medium extravasation in acute stroke patients following intra-arterial revascularization [11]. Previous studies have reviewed DECT applications within the abdomen and gastrointestinal tract using virtual non-contrast (VNC) [12,13] and virtual monoenergetic (VM) reconstructions [14]. The purpose of this systematic review was to outline the clinical DECT applications of iodine quantification (IQ) in the gastrointestinal tract.

### DECT and Iodine Quantification

DECT allows for a range of image series to be obtained aside from conventional monochromatic images. The post-processing techniques can be separated into two groups. First, VM images provide reconstructions of specific kiloelectron volt (KeV). This allows for iodine contrast enhancement and artifact reduction reconstructions, which have been used in cardiac, pulmonary and abdominal vascular imaging [15,16,17,18], oncological imaging [19,20,21] and metal artifact reduction [22,23]. The second type of reconstructions are linked to material decomposition, i.e., differentiation of tissues based on atomic composition, which enables the separation of iodine contrast for VNC reconstructions [24,25]. VNC has shown to be comparable to conventional non-contrast images, allowing for a reduction in radiation dosage as a true non-contrast scan in certain cases can be omitted [26,27].

Pixel attenuation in CT and DECT is defined by the Hounsfield Unit (HU). In comparison with conventional CT, DECT relies on the varying attenuation of different materials, when exposed to photons of different energies. For instance, with tube voltages of 80 and 140 peak kilovoltage (kVp), the HU values for iodinated contrast are 600 and 300, respectively, resulting in an iodine ratio of two. For calcified bone this ratio is 1.5 (800 HU/533 HU). The variation of ratios can be used to differentiate between iodine and other constituents of the human body such as parenchymal tissue, fat and bone. Iodine concentration (IC) is a feature of IQ and can be derived and is most commonly defined by the units mg/mL, mg/cm^3^ or μg/mL. Alongside IC, features such as Z-effective number and the slope of the HU curve have likewise been used as a surrogate measure for IQ [28,29]. These measurements can be applied in the different scan phases, e.g., arterial (IC-A), venous (IC-V) or delayed phase (IC-D). Multiple studies investigating IQ have included normalized IC (nIC) as an outcome measure [30,31,32], which is calculated based on the equation nIC = IC_lesion_/IC_reference_ (the reference most commonly being the aorta). The main point of including nIC is to reduce technical or physiological variabilities in iodine load within the tissue of interest due to varying cardiac output and phase times.

## 2. Materials and Methods

This systematic review was performed using the preferred reporting items for systemic reviews and meta-analyses (PRISMA) guidelines [33]. The review protocol was not published in advance.

### 2.1. Search Strategy

The literature search was conducted on the 13th of May 2020 using three databases: Pubmed, EMBASE and Cochrane Library. The search was restricted to peer-reviewed publications of original research using the population, intervention, comparison and outcome approach (PICO) model [34]: the patient group had gastrointestinal-related conditions; the intervention consisted of DECT examination from which IQ was measured; the comparison was to other verified methods of evaluation for the given condition, such as conventional CT, surgical findings, pathology, biochemistry, etc.; and the main outcome was establishing whether DECT examination and IQ measurements correlate with verified measures of evaluation.

The search thread in PubMed contained two aspects, consisting of MeSH (Medical subject headings) terms as well as text words (in the title and/or abstract), which were combined using “OR” and “AND”.

The first aspect focused on DECT, including the MeSH term “Radiography, Dual-Energy Scanned Projection” as well as a combination of the MeSH term “Tomography, X-Ray Computed/methods” with the text word “dual energy”. Additional text words were added including “DECT”, “Dual-energy CT”, “Spectral CT” and “Dual-energy Computed Tomography”.

The second aspect focused on gastrointestinal diseases and included the MeSH terms “Gastrointestinal diseases” and “Gastrointestinal tract”. Additional truncated text words included “gastrointestinal tract”, “GI-tract”, “esophagus”, “gastric”, “Stomach”, “duodenum”, “jejunum”, “ileum”, “bowel”, “intestine”, “Colon” and “Rectum”.

The Cochrane Library search was conducted with the Pubmed search string using two aspects combining MeSH terms as well as free text words. The function “explode all trees” was applied to all MeSH terms as well as “include word variations” to all free text words.

The EMBASE search was conducted in a similar fashion using the EMTREE terms “dual energy computed tomography”, “gastrointestinal disease” and “gastrointestinal tract” in combination with identical text words.

### 2.2. Study Selection

Study selection was conducted on the online platform covidence.org. The initial selection was based on study titles and abstracts by two independent assessors (J.J.X. and M.T.). The first assessor was a PhD fellow with one year of clinical radiology experience, and the second assessor was a radiologist specialized in interventional radiology with 10 years of clinical radiology experience. Selection of studies was carried out separately. Studies were selected based on the presence of search terms in abstract and title, and the full text was retrieved for studies that were eligible or possibly eligible, and then independently screened by the assessors. Discrepancies regarding potential eligibility and inclusion were resolved by consensus.

Eligibility for this systematic review included two inclusion criteria: DECT of the gastrointestinal tract and DECT including or focusing on IQ as an outcome measure. Exclusion criteria were as follows: study population <10 patients, phantom-only or animal-only studies, language other than English, review articles, case reports or editor’s letters.

### 2.3. Quality Assessment

Potential risk of bias was assessed using the QUADAS-2 tool [35]. The four domains—patient selection, index test, reference test, and patient flow—were assessed for potential risks of bias and applicability concerns (Figure S1). The rating score was either low (☺), high (☹), or unclear (?).

## 3. Results

**Figure 1:**
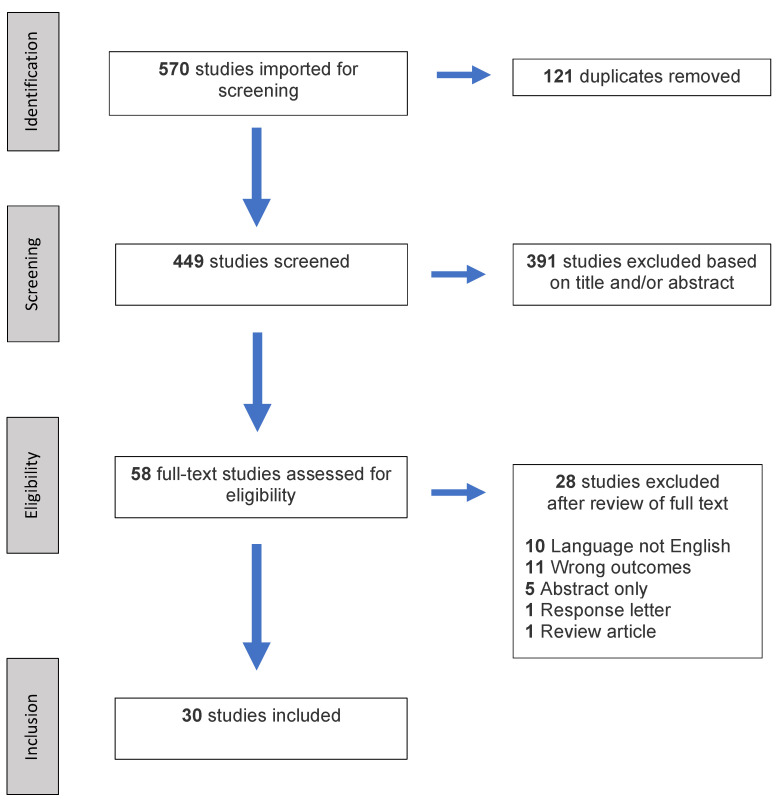
PRISMA flowchart of the literature search and study selection.

The primary search thread identified 570 studies for inclusion in the methodological review. After duplicates were removed, 449 studies were screened based on set inclusion and exclusion criteria resulting in the inclusion of 30 studies (Figure 1). The 30 included studies involved 1778 patients with a mean population size of 59 patients (range: 11–162). Twelve studies involved gastric tumors, eight studies on colorectal tumors, four studies on Crohn’s disease, and six on miscellaneous applications. The latter included peristalsis-related streak artifact reduction, acute bowel ischemia, esophageal cancer and gastrointestinal stromal tumor (GIST) risk stratification.

### 3.1. Gastric Tumors

Four studies demonstrated a significant difference in IQ when comparing poorly differentiated and moderately/well-differentiated adenocarcinoma with region of interest (ROI) placement on a solid tumor mass. Outcome measures of nIC in arterial phase (nIC-A) and nIC in venous phase (nIC-V) were used in all studies, with one study also including IC in arterial phase (IC-A) and IC in venous phase (IC-V) [36,37,38,39]. Among these four studies, there seemed to be no correlation with TNM classification of malignant tumors. However, studies investigating IC in perigastric adipose tissue among gastric cancer patients [40,41,42,43] found a significant difference between patients with (T4) and without serosal invasion (T1-T3). Additionally, a study by Cheng et al. [44] noted a significant difference between early (confined to mucosa/submucosa) vs. advanced gastric cancer (invasion of the submucosa) in nIC-V and nIC in delayed phase (nIC-D) with ROI placement on the gastric mass. One study by Tang et al. [45] demonstrated that the relative reduction in IC (defined by [IC_after_ − IC_before_]/IC_before_ × 100%) following neoadjuvant chemotherapy correlated well with the histopathological regression. Furthermore, two studies by Liu et al. [46] and Meng et al. [47] found a significant difference in IC between GIST and gastric schwannomas, as well as gastric cancer and normal gastric mucosa or gastric inflammation (Studies shown in Table 1).

### 3.2. Colorectal Tumors

Two studies demonstrated a significant difference in IC between poorly and moderate/well-differentiated colorectal cancer [48,49]. Chuang-bo et al. [49] only found a significant difference during the arterial phase, while Gong et al. [48] reported a significant difference in both the arterial and the venous phase. Similar to the gastric cancer studies [42,45], a positive correlation between IC and pathological grading of rectal cancer prior and following chemoradiotherapy treatment was reported [50]. However, there is a discrepancy of whether IC may be used in differentiating between malignant and benign colorectal tumors. While Al-Najami et al. [51] found no significant differences in IC when comparing malignant with benign rectal tumors, Sun et al. [52] demonstrated significant differences in IC between colonic adenomas and adenocarcinomas. Additionally, three studies determined positive correlations with other paraclinical measures such as perfusion computed tomography (CT) parameters (blood flow, blood volume, permeability, mean transit time), immunohistochemical evaluation of Ki-67 and HIF-1α levels as well as microsatellite stability and instability [53,54,55] (Studies shown in Table 2).

### 3.3. Crohn’s Disease

Two of four DECT enterography studies concerning Crohn’s disease used Crohn’s disease activity index (CDAI) as reference [56,57], and two studies used endoscopy, biochemistry and clinical symptoms as reference [30,58]. The outcome measure included nIC-V for all studies except one study by Kim et al. [56], which only included IC-V. ROI placements were set on either iodine maps or conventional images covering the most enhanced areas. All studies found a strong correlation between IC measurements and CDAI or endoscopy findings (Studies shown in Table 3).

### 3.4. Miscellaneous Applications

Similar to gastric and colorectal cancer, Ge et al. [59] demonstrated a positive correlation among esophageal cancer patients between nIC and response following chemoradiotherapy based on response criteria in solid tumors (RECIST) criteria. Unlike the studies in the gastric and colorectal group [36,48,49], two studies [31,60] suggested that IC/nIC can differentiate between specific cancer subtypes such as squamous cell carcinoma and adenocarcinoma in the gastroesophageal junction, as well as discriminate between small bowel adenocarcinoma and primary small intestine lymphoma. Additionally, Zhang et al. [32] found a significant difference in IC between high- and moderate/low-risk GIST patients based on GIST recurrence risk stratification criteria [61].

There were two outlying studies in this group. The first study by Lourenco et al. [62] demonstrated a significant reduction in IC in bowel segments suffering from acute bowel ischemia. The second study by Winklhofer et al. [63] demonstrated that peristalsis-related streak artifacts can be reduced using iodine maps (Studies shown in Table 4).

## 4. Discussion

This review represents a heterogenous group of studies with a major focus on gastrointestinal cancer evaluation and diagnoses. The main findings include positive correlations between IC and degree of cell differentiation in adenocarcinomas, treatment response following chemo- or chemoradiotherapy, Crohn’s disease activity and differentiation of T1-3 vs. T4 stage gastric cancer (Table 1, Table 2 and Table 3).

One of the most convincing DECT applications for gastrointestinal imaging is probably related to the differentiation of T1–3 vs. T4 stage gastric cancer based on IC measurements in the perigastric adipose tissue as seen in Table 1 [40,41,42,43]. An additional finding was the correlation between IC and varying degrees of cell differentiation in adenocarcinoma. Two colorectal cancer and five gastric adenocarcinoma studies [36,37,38,39,41,48,49] demonstrated an overall positive correlation between IC/nIC and the degree of differentiation, when the ROI was placed within the tumor mass as shown in Table 1 and Table 2. These studies had an overall low risk of bias (Figure S1). Only one study by Xie et al. [41] found no significant difference in the degree of differentiation, with *p*-values of 0.06, 0.07 and 0.09 in nIC-A, nIC-V and nIV-D, respectively. Aside from the IC measurements in perigastric adipose tissue, several studies have also suggested that IC measurements in lymph nodes may be used to discriminate metastatic from non-metastatic lymph nodes relating to gastric as well as colorectal cancers [36,64]. For gastric cancers, the evidence suggests that IQ with ROI placement on solid tumor correlates well with degree of differentiation, while IC measurements with ROI placement in the perigastric adipose tissue correlates with serosal invasion [40,42].

Four studies [42,45,50,59] investigated the correlation between IC measurements with pre- and post-chemo or chemoradiotherapy treatment (three studies = neoadjuvant, one study = curative). IC was correlated to pathological findings, and all studies found a positive correlation between IC measurements and pathological response or non-response, suggesting that IQ may be used in monitoring treatment response. These findings stand in contrast to a study by Mazzei et al. [65], which identified no significant differences between tumor HU attenuation and different tumor regression grades among gastric cancer patients prior to and following neoadjuvant chemotherapy. Similarly, IQ may also aid in the monitoring of disease activity in the relatively homogenous group of Crohn’s disease patients. Three studies [30,56,57], with a relatively low risk of bias (Figure S1), found significant correlation between IC measurements and various validated methods of evaluating disease activity such as CDAI, endoscopy and biochemistry as shown in Table 3.

An inconsistency, aside from the varying study focuses, seems to be the chosen outcome measures. Of the 30 studies, 12 studies included only IC measurements, 12 studies included only nIC measurements, and eight studies included IC as well as nIC measurements. None of the included studies detailed the rationale behind including or excluding nIC as an outcome measure. The applications and benefits of IC normalization remain unclear. Logically, nIC may reduce variability in cases of varying contrast administration times, flow rates and varying iodine contrast medium concentrations, as the measurement in tissue is referenced to a highly iodinated structure (e.g., the aorta). One study by Patel et al. [66] focused on vascular vs. non-vascular renal lesions and whether normalization of IC to the aorta could reduce inter-manufacturer threshold variability. The study found that nIC reduces the inter-manufacturer variability in IC measurements with no significant inter-manufacturer difference in nIC (*p >* 0.05), but a significant difference in absolute IC (*p <* 0.05). However, the study did not assess the effects of nIC on minimizing patient (e.g., reduced cardiac output) or technical variabilities (i.e., timing of contrast medium administration, total iodine, flow rate).

Additionally, IC normalization reference points varied among included studies, with the most common reference being the aorta. However, multiple studies have used other arteries such as the external iliac artery [53,55] as well as the psoas muscle as reference [48], making comparison of nIC between studies a challenge.

IQ is only one of several possible reconstructive measures in DECT image acquisition. Alongside IQ generated through the ROI placement, several other quantitative parameters may be measured at the same time, such as Z-effective number and the slope of the spectral HU curve. In the scope of this review, these parameters should in theory not differ significantly from each other, as they are all directly or indirectly an expression of the IC within the ROI. This assumption aligned well with the outcomes of all studies including Z-effective and/or slope of the HU curve (*n* = 10). Of these, only one study, by Al-Najami et al. [51] with a sample size of 16 patients, found a significant difference in the effective Z number, but not in the IC for the differentiation of malignant vs. benign rectal tumors.

Several potential clinical implications of IQ have been suggested. In the case of gastric and colon cancers, studies have suggested that IQ may improve the preoperative diagnosis and evaluation [34,38]. Chuang-bo et al. [49] reported increased sensitivity and specificity for well-differentiated vs. poorly differentiated carcinoma using IQ, when compared with conventional CT images at 70 KeV (*p <* 0.05). Additionally, several studies have suggested that IQ may be an alternative method to evaluate chemo- or chemoradiotherapy treatment response [50]. Ge et al. [59] reported a significant difference in nIC (*p <* 0.05) when comparing the effective treatment group with the ineffective group using RECIST as reference. A study by Uhrig et al. [67] suggested that IQ using DECT may be a complementary method to RECIST, as RECIST only accounts for size reduction of the tumor due to the cytotoxic effects of chemotherapy. Targeted therapies interfere with various biological pathways and may cause tumor necrosis or hemorrhage [68,69], rendering an underestimation of the treatment response according to RECIST criteria. Compared with conventional CT, IQ using DECT overcomes these drawbacks, as IC measurements are not affected by tissue modifications, such as necrosis or hemorrhage, but are purely a representation of vascularized tumor tissue. Other modalities such as magnetic resonance imaging (MRI) and fluorodeoxyglucose positron emission tomography (FDG-PET) have proven to be beneficial in monitoring treatment response [70,71,72]; however, there is no established golden standard, and the examination costs associated with DECT are substantially lower compared to MRI and FDG-PET.

CT colonography also seems to benefit from IQ and DECT image acquisition improving diagnostic accuracy for colorectal cancer screening [52] when comparing conventional CT colonography with DECT colonography. Lastly, IQ may be a convenient and reproducible measure in the evaluation of Crohn’s disease [30], with a study by Kim et al. [54] reporting significant correlation (*r* = 0.74) between IC and CDAI. However, in the case of Crohn’s disease, the patient age and radiation dosage should be considered.

IQ by means of ROI placement has its limitations. In the case of acute bowel ischemia, ROI placement may be challenging in times of bowel wall thinning due to, e.g., arterial occlusion [73], and mucosal enhancements in arterial occlusive and non-occlusive ischemia are different in the presence or absence of a reperfusion event [74]. In certain cases, qualitative reconstructions such as virtual monoenergetic or iodine mapping have shown to increase conspicuity and confidence in the diagnosis of acute bowel ischemia [62,75].

There are several limitations to this study. First, there is an obvious heterogeneity in the studies, preventing meta-analysis in comparing IQ with specific correlation measures. Among the different subgroups, the objectives of the studies varied vastly from differentiating between cancer subtypes to evaluating the response following neoadjuvant chemotherapy. Second, the median patient sample size is quite small considering the heterogeneity of the studies. Several studies included no more than 20 patients, making statistical significance questionable.

Third, there are considerable inconsistencies in terms of DECT image acquisition parameters across studies. Variables include different concentrations of iodinated contrast agents, contrast flow rates, total iodine, phase times for all three phases as well as different CT scanners with different postprocessing software, which has shown to have varying accuracies in regard to iodine measurements [76]. These inconsistencies may also be troublesome for future meta-analyses, as the outcome measure relies on an input measure, which for various reasons will vary.

Future studies in this field should include larger sample sizes to decrease the margin of error. In addition, the potential applications of DECT and IQ relating to small bowel pathologies such as bowel ischemia are poorly elucidated and warrant further investigation. Additionally, the use of nIC vs. IC should be investigated further to assess whether nIC provides benefits in terms of reducing variability among patients with decreased cardiac output, varying technical factors such as flow rate, total iodine and varying phase times.

## 5. Conclusions

Despite the heterogeneity of this systematic review, certain applications within the GI tract are better elucidated than others. Some of the promising applications of IQ include differentiating between gastric cancers with and without serosal invasion, identifying the degree of differentiation in adenocarcinomas, monitoring of chemo- or chemoradiotherapy treatment response and Crohn’s disease activity.

## Figures and Tables

**Table 1:** Studies investigating applications related to gastric tumors.

Author	Year	Focus	Population	DECT Scanner	kV Range	Contrast	Flow Rate	Total Iodine	ROI Placement	Reference	Phase	Normalization	Outcome Measure	Findings
Pan [36]	2013	Degree of differentiation gastric cancer	96	Discovery CT750 HD, GE Healthcare	80/140	Ultravist 300 mg I/mL	3 mL/s	85–110 mL	Lesion and the normal gastric wall.	Pathology	AP/VP	Aorta	nIC-A, nIC-V	Significant difference between well-differentiated and poorly differentiated adenocarcinoma in both phases (nIC-A: *p* < 0.02, nIC-V: *p* < 0.05). Significant difference between metastatic and non-metastatic lymph nodes. nIC had no correlation with cancer subtypes.
Tang [45]	2015	Evaluating the response of gastric carcinomas to neoadjuvant chemotherapy	20	Discovery CT750 HD, GE Healthcare	80/140	Omnipaque 300 mg I/mL	3.5 mL/s	1.5 mL/kg	N/A	Pathology	AP/VP	N/A	IC-A, IC-V,	% decrease in IC-A was significantly different in good response group vs. poor response group (*p* = 0.012)
Yang [40]	2015	Assessment of IC in perigastric fat in gastric cancer patients with and without serosal invasion (Stage T4a)	54	SOMATOM Definition Flash, Siemens Healthcare	100/140	Omnipaque 300 mg I/mL	3.0 mL/s	2 mL/kg	Perigastric fat adjacent to tumor	Pathology	AP/VP	N/A	IC-A, IC-V	Significant difference between patients with and without serosal invasion in both phases (*p* < 0.001)
Liang [37]	2017	Correlation with clinicopathologically determined prognostic factors in gastric adenocarcinoma (TNM, MVD)	34	Discovery CT750 HD, GE Healthcare	N/A	Optiray 320 mg I/mL	3 mL/s	1.5 mL/kg	Area that encompassed the entire tumor, away from any peripheral fat and necrotic areas.	Pathology	AP/VP	Aorta	nIC-A, nIC-V	Significant difference between moderately and poorly differentiated adenocarcinoma (nIC-A: *p* = 0.005, nIC-V: *p* = 0.013). Positive correlation between nIC and MVD (nIC-A: *r* = 0.423, nIC-V: *r* = 0.542). No correlation with lymphatic metastasis or TNM stage (nIC-A: *r* = 0.119, nIC-V: *r* = 0.097)
Liu [46]	2017	Value of DECT in Gastric schwanomma and GIST	12	Discovery CT750 HD, GE Healthcare	80/140	Omnipaque 300 mg I/mL	3–4 mL/s	1.0 mL/kg	Tumor; avoiding necrosis and cystic areas, calcification, and larger vessels	Pathology	AP/VP	N/A	IC-A, IC-V	Significant difference in IC betweengastric schwanommas and GIST (*p* < 0.001)
Chen [38]	2017	Correlation with MVD in gastric cancer patients	34	Discovery CT750 HD, GE Healthcare	80/140	Omnipaque 350 mg I/mL	2.5–4.5 mL/s	60–110 mL	Lesion; avoiding artifacts, necrosis, and vessels	Pathology	AP/VP	Aorta	nIC-A, nIC-V	Significant difference between well and poorly differentiated adenocarcinoma (nIC-A: *p* < 0.003, nIC-V: *p* < 0.001). Positive correlation between nIC and MVD (nIC-A: *r* = 0.423, nIC-V: *r* = 0.606).
Meng [47]	2017	Differentiation between malignant and benign gastric lesions (Cancer, Inflammation, normal)	161	Discovery CT750 HD, GE Healthcare	80/140	Ultravist 370 mg I/mL	3–4 mL/s	1.0 mL/kg	Lesion; avoiding cystic, necrosis, and hemorrhage	Pathology	AP/VP	Aorta	nIC-A, nIC-V, IC-A, IC-V	nIC and IC in gastric cancer differed significantly from normal mucosa and gastric inflammation (*p <* 0.05, aside from nIC-A: *p* = 0.116)
Xie [41]	2018	T and N staging of gastric cancer	71	SOMATOM Definition Flash, Siemens Healthcare	100/140	Omnipaque 350 mg I/mL	2.5–3 mL/s	70 mL	Tumor and extraserosal fat	Pathology	AP/VP/DP	Aorta	nIC-A, nIC-V, nIC-D	Significant difference between T3 and T4 in extraserosal fat in arterial and dealyed phase (nIC-A: *p* = 0.004, nIC-D: *p* = 0.001). No significant findings between differentiated vs. Undifferetiated adenocarcinoma (nIC-A: *p* = 0.06, nIC-V: *p* = 0.07, nIC-D: *p* = 0.09) with ROI placement on tumor
Yang [42]	2018	IC in perigastric adipose tissue in the assessment of Serosal Invasion in Patients with Gastric Cancer after Neoadjuvant Chemotherapy	43	SOMATOM Definition Flash, Siemens Healthcare	100/140	Omnipaque 300 mg I/mL	3.0 mL/s	2 mL/kg	Perigastric adipose tissue without blood vessels or other tissues	Pathology	AP/VP	Aorta	nIC-V, IC-V	Significant difference between patients with and without serosal invasion pre- and post neoadjuvant chemotherapy (*p <* 0.05) aside from nIC in patients with serosal invasion prior to chemotherapy (*p* = 0.10)
Cheng [44]	2018	Correlation with Ki-67 protein level expression in advanced & early Gastric cancer	162	Discovery CT750 HD, GE Healthcare	80/140	Iopamidol 370 mg I/mL	3.0 mL/s	1.8 mL/kg	Solid tumor; avoiding necrotic and fat areas	Pathology	AP/VP/DP	Aorta	nIC-A, nIC-V, nIC-D, IC-A, IC-V, IC-D	Significant difference between early (confined to mucosa/submucosa) vs. advanced gastric cancer (invasion of the submucosa) in nIC-V/D (*p* = 0.002, *p* = 0.000) and IC-V/D (*p* = 0.029, *p* = 0.002). Ki/67 correlates well with nIC-V/D (*r* = 0.753, *r* = *0.745*) and IC-V/D (*r* = 0.818, *r* = 0.730)
Li [39]	2018	Discrimination between benign and malignant & correlation to degree of differention	87	Discovery CT750 HD, GE Healthcare	80/140	Ultravist 370 mg I/mL	3.0 mL/s	1.5 mL/kg	Solid part of the tumor; avoiding peripheral fat, visible vessel, calcification and cystic/necrotic areas.	Pathology	AP/VP	Aorta	nIC-A, nIC-V, IC-A, IC-V	Significant difference between well-differentiated and poorly differentiated adenocarcinoma in all phases (*p <* 0.0001, except nIC-A: *p* = 0.0445).
Küpeli [43]	2019	IC measurements in the perigastric fat and its’ correlation with gastric cancer TNM staging	41	Aquilion, Toshiba Medical Systems	80/130	(nonionic contrast agent)	4.0 mL/s	2 mL/kg	Normal gastric tissue, tumor and perigastric fat	Pathology	AP/VP	N/A	IC-A, IC-V	Significant difference in IC-A (*p <* 0.001) and IC-V (*p <* 0.001) between patients with serosal invasion (T4) vs. Absent(T1-3)

Studies listed chronologically according to publication year. Abbreviations: arterial phase (AP), venous phase (VP), delayed phase (DP), iodine concentration (IC), iodine concentration in arterial phase (IC-A), iodine concentration in venous phase (IC-V), iodine concentration in delayed phase (IC-D), normalized iodine concentration in arterial phase (nIC-A), normalized iodine concentration in venous phase (nIC-V), normalized iodine concentration in delayed phase (nIC-D), region of interest (ROI), gastrointestinal stromal tumor (GIST), microvascular density (MVD).

**Table 2:** Studies investigating applications related to colorectal tumors.

Author	Year	Focus	Population	DECT Scanner	kVp Range	Contrast	Flow Rate	Total Iodine	ROI Placement	Reference	Phase	Normalization	Outcome Measure	Findings
Gong [48]	2016	Colorectal cancer differentiation degree	81	Discovery CT750 HD, GE Healthcare	N/A	Iopamidol 370 mg I/mL	3.5 mL/s	1.8 mL/kg	Solid tumor regions avoiding areas with obvious features of cystic or necrotic change	Pathology	VP	Psoas muscle	nIC-A, nIC-V, IC-A, IC-V	Significant difference between well and moderately differentiated vs. Poorly differentiated colonic adenocarcinoma in all phases (*p* = 0.000)
Al-Najami [50]	2017	Regression assessment in rectal cancer patients following neoadjuvant chemoradiotherapy treatment	11	Discovery CT750 HD, GE Healthcare	80/140	Omnipaque 300 mg I/mL	3 mL/s	1 mL/kg	Tumor; based on a macroscopic evaluation of the most representative images of associated MRI scan	Pathology (RCRG)	N/A	N/A	IC	Significant difference in IC in partial and complete response group following neoadjuvant chemoradiotherapy treatment (*p <* 0.05)
Chaung-bo [49]	2017	Colon cancer differentiation degree	47	Discovery CT750 HD, GE Healthcare	N/A	Iohexol 300 mg I/mL	3–4 mL/s	0.8–1 mL/kg	Tumor tissue; avoiding areas of necrosis, calcification, and artifacts caused by the gas and liquid interface	Pathology	AP/VP	Aorta or iliac artery	nIC-A, nIC-V, IC-A, IC-V	Significant difference in nIC-A (*p* = 0.02) and IC-A (*p* = 0.001) between poorly and well-dffierentiated colon cancer
Fan [53]	2017	Correlation with Ki-67 and HIF-1α in rectal cancer	80	Discovery CT750 HD, GE Healthcare	80/140	Omnipaque 300 mg I/mL	2.5 mL/s	1.2 mL/kg	Solid tumor regions avoiding areas with obvious features of cystic or necrotic change	Ki-67 and HIF-1α	70 s	External Iliac artery	nIC-V	Postively correlated with Ki-67 value (*r* = 0.344, *p* = 0.002) and HIF-1α levels (*r* = 0.598, *p* < 0.001) in rectal cancer patients
Sun [52]	2018	Accuracy of Combined CT Colonography and DECT iodine mapping for Detecting Colorectal masses	28	SOMATOM Definition Flash, Siemens Healthcare	100/140	Omnipaque 350 mg I/mL	4.0 mL/s	60 mL	Tumor	Optical colonoscopy and Pathology	4 s (post bolus tracking)	N/A	IC-A	Significant difference in IC between stool and colonic neoplasia (*p* < 0.01). Significant difference nIC between colonic adenomas and adenocarcinomas (*p <* 0.01)
Kang [54]	2018	Correlation with perfusion CT parameters in colorectal cancer	41	SOMATOM Definition Flash, Siemens Healthcare	80/140	Bonorex 350 mg I/mL	4–5 mL/s	1.125 mL/kg	Tumor	Perfusion CT measurements (Blood flow, blood volume, permeability, mean transit time)	50 s	Aorta and inferior vena cava	nIC-V, IC-V	IC-V correlates with some perfusion CT parameteres (Blood volume: *r* = 0.32, *p* = 0.04; permeability: *r* = 0.34, *p* = 0.03; mean transit time: *r =* −0.38, *p* = 0.02)
Wu [55]	2019	Discriminating MSI from MSS in human colorectal cancer	114	Discovery CT750 HD, GE Healthcare	80/140	Omnipaque 300 mg I/mL	3–3.5 mL/s	1.2 mL/kg	Solid tumor; avoiding bleeding, necrosis, and cystic portions	Pathology (Immunohistochemical staining)	AP/VP/DP	External Iliac artery	nIC-A, nIC-V, nIC-D	Significant difference in nIC between MSS and MSI in all phases (*p <* 0.001)
Al-Najami [51]	2019	Differentiation between malignant and benign rectal tumors	16	Discovery CT750 HD, GE Healthcare	80/140	Omnipaque 300 mg I/mL	3 mL/s	1 mL/kg	Tumor; most representative areas of evident tumor tissue	Pathology	N/A	N/A	IC	Z-effective was significant between malignant * and benign group (*p* = 0.03), however IC was nonsignificant (*p* > 0.05)

Studies listed chronologically according to publication year. * Defined as T1 or T2 stage. Abbreviations: Arterial phase (AP), venous phase (VP), delayed phase (DP), iodine concentration (IC), iodine concentration in arterial phase (IC-A), iodine concentration in venous phase (IC-V), normalized iodine concentration in arterial phase (nIC-A), normalized iodine concentration in venous phase (nIC-V), normalized iodine concentration in delayed phase (nIC-D), region of interest (ROI), microsatellite stability (MSS), microsatellite instability (MSI), rectal cancer regression grade (RCRG).

**Table 3:** Studies investigating applications related to Crohn’s disease.

Author	Year	Focus	Population	kVp Range	DECT Scanner	Contrast	Flow Rate	Total Iodine	ROI Placement	Reference	Phase	Normalization	Outcome Measure	Findings:
Peng [30]	2016	Disease activity in ileocolonic crohns disease	50	80/140	Discovery CT750 HD, GE Healthcare	Iopamidol 370 mg I/mL	4 mL/s	1.5 mL/kg	Placed on iodine concentration maps and encompassed the high-enhancing areas	Endoscopy (Simple Endoscopic Score for Crohn’s Disease)	45 s	Artery (not specified)	nIC-V	Significant differences in nIC-V between endoscopic normal and mild (*p* = 0.002) as well as mild and severe lesions (*p* < 0.001)
Kim [56]	2018	Correlation with disease activity index	39	120	IQon Spectral CT, Philips Healthcare	Iohexol, 350 mg I/mL	3–5 mL/s	1.6 mL/kg	Bowel wall with strongest enhancement on iodine concentration maps	Crohn’s disease activity index (CDAI)	VP	N/A	IC-V	Iodine concentrations correlates well with CDAI score (*r* = 0.744, *p* < 0.001)
DeKock [58]	2019	Distinguishing normal small bowel from active inflammatory crohns	40	80/140	SOMATOM Definition Flash, Siemens Healthcare	Visipaque, GE healthcare	3.5 mL/s	100 mL	Normal bowel wall = ROI over entire bowel wall. Crohns = ROI placed on mucosa (brightest area)	Endoscopy, biochemistry and clinical symptoms	70 s	Aorta	nIC-V, IC-V	Significant difference between disease and control group (*p* < 0.001)
Dane [57]	2020	Correlation with disease activity	22	80/150	SOMATOM FORCE, Siemens Healthcare	Ultravist 300 mg I/mL	3–4 mL/s	1.5 mL/kg	Brightest involved bowel wall segment	Crohn’s disease activity index (CDAI)	60 s	Aorta	IC-V (Min, max and weighted average)	The ICmax and ICmin of affected bowel differed significantly from normal bowel (*p* < 0.0001)

Studies listed chronologically according to the publication year. Abbreviations: venous phase (VP), iodine concentration in venous phase (IC-V), normalized iodine concentration in venous phase (nIC-V), region of interest (ROI), Crohn’s disease activity index (CDAI).

**Table 4:** Studies investigating miscellaneous applications.

Author	Year	Focus	Population	DECT Scanner	kVp Range	Contrast	Flow Rate	Total Iodine	ROI Placement	Reference	Phase	Normalization	Outcome Measure	Findings:
Winklhofer [63]	2016	Reduction of peristalsis-related gastrointestinal streak artifacts	100	Discovery CT750 HD, GE Healthcare	80/140	N/A	N/A	N/A	The most visibly bright area of streak artifact in the 70 keV axial images	ROI measurements in streak artifacts vs. non artifact in 70 keV, 120 keV and water (iodine)	VP	N/A	N/A	ROI measurements in areas with and without streak artifcts were non-significant in iodine/water (*p* = 0.088) compared to monoenergetic images and water/iodine (*p* < 0.001). Streak artifacts are reduced in iodine/water images
Lourenco [62]	2018	Applications in Acute Bowel ischemia	60	SOMATOM Definition Flash, Siemens Healthcare	100/140	Omnipaque 350 mg I/mL	3.5 mL/s	90 mL	Ischemic and normalbowel	Electronic medical record, procedrual and pathology reports	VP	N/A	IC-V	65% reduction in IC among patients with confirmed ischemia *
Ge [59]	2018	Iodine concentrations in esophageal cancer before and after chemoradiotherapy	45	SOMATOM Definition Flash, Siemens Healthcare	100/140	Iohexol 300 mg I/mL	3 mL/s	70 mL	Tumor; avoiding tumor margins and necrotic areas	RECIST criteria	AP/VP	Aorta	nIC-A, nIC-V	Significantly lower nIC-A and nIC-V in effective group vs. ineffective group post-chemoradiotherapy (*p < 0.05*)
Zhou [31]	2019	Differentiation between squamous cell carcinoma and adenocarcinoma in the gastroesophageal junction	61	Discovery CT750 HD, GE Healthcare	80/140	Iobitrido 350 mg I/mL	3 mL/s	1.5 mL/kg	Around the entire lesion	Pathology	AP/VP	Arota	nIC-A, nIC-V (nIC difference, nIC ratio)	Significant difference between squamous cell carcinoma and adenocarcinoma in both phases (nIC-A: *p* = 0.02, nIC-V: *p* = 0.00)
Yang [60]	2019	Differetiation between small bowel adenocarcinoma and primary small intestinal lymphoma	42	Discovery CT750 HD, GE Healthcare	80/140	Omnipaque 300 mg I/mL	3–4 mL/s	0.8–1.0 mL/kg	Tumor; avoiding focal necrosis, calcification, and blood vessels	Pathology	AP/VP	Aorta	nIC-A, nIC-V, IC-A, IC-V	Significant difference between small bowel adenocarcinoma and primary small intestinal lymphoma in nIC-A (*p* = 0.001), nIC-V (*p* = 0.002) and IC-A (*p* = 0.003)
Zhang [32]	2019	Value of IC paramenters in gastrointestinal stromal tumor risk stratification	86	Discovery CT750 HD, GE Healthcare	N/A	Omnipaque 300 mg I/mL	3.5–4.0 mL/s	1.2 mL/kg	Primary lesion and normal intestinal wall	Pathology (GIST recurrence risk stratification criteria)	AP/VP/DP	Aorta	nIC-A, nIC-V, nIC-D	Significant difference between high risk and intermediate/low risk GIST patients in all phases (*p* < 0.001)

Studies listed chronologically according to publication year. * *p*-value was not specified. Abbreviations: arterial phase (AP), venous phase (VP), delayed phase (DP), iodine concentration in arterial phase (IC-A), iodine concentration in venous phase (IC-V), normalized iodine concentration in arterial phase (nIC-A), normalized iodine concentration in venous phase (nIC-V), normalized iodine concentration in delayed phase (nIC-D), gastrointestinal stromal tumor (GIST), region of interest (ROI), response evaluation criteria in solid tumors (RECIST).

## Supplementary Materials

The following are available online at https://www.mdpi.com/2075-4418/10/10/814/s1, Figure S1. Risk of bias and application concern assessment using the QUADAS-2 tool. Studies are listed chronologically based on publication year. Symbols defining the following: low (☺), high (☹), or unclear (?).

## Abbreviations

AP	Arterial phase
CDAI	Crohn’s disease activity index
CT	Computed tomography
DECT	Dual-energy Computed Tomography
DP	Delayed phase
GIST	Gastrointestinal stromal tumor
IC	Iodine concentration
IC-A	Iodine concentration in arterial phase
IC-V	Iodine concentration in venous phase
IQ	Iodine quantification
KeV	Kiloelectron volt
kVp	Peak kilovoltage
HU	Hounsfield unit
MDCT	Multi-detector computed tomography
MeSH	Medical subject heading
MSI	Microsatellite instability
MSS	Microsatellite stability
MVD	Microvascular density
nIC	Normalized iodine concentration
nIC-A	Normalized iodine concentration in arterial phase
nIC-V	Normalized iodine concentration in venous phase
nIC-D	Normalized iodine concentration in delayed phase
PRISMA	Preferred reporting items for systematic reviews and meta-analyses
QUADAS-2	Quality Assessment of Diagnostic Accuracy Studies 2
RCRG	Rectal cancer regression grade
RECIST	Response evaluation criteria in solid tumors
ROI	Region of interest
VM	Virtual monoenergetic
VP	Venous phase
VNC	Virtual non-contrast
