# Supplementary material for: Gastrointestinal Applications of Iodine Quantification Using Dual-Energy CT: A Systematic Review
Source: Diagnostics (Basel). 2020 Oct 13;10(10):814. doi: 10.3390/diagnostics10100814 (PMC7602017; doi:10.3390/diagnostics10100814)
Supplement: Supplementary file 1 [file diagnostics-10-00814-s001.pdf]

diagnostics

MDPI

Study	Risk of Bias					Applicability Concerns		
	PATIENT SELECTION	INDEX TEST	REFERENCE STANDARD	FLOW AND TIMING		FLOW AND TIMING	INDEX TEST	REFERENCE STANDARD
Pan 2013 [36]	☺	☺	?	☺		☺	☺	☺
Tang 2015 [45]	☺	☺	?	☺		☺	☺	☺
Yang 2015 [40]	☺	☺	☺	☺		☺	☺	☺
Peng 2016 [30]	☺	☺	☺	☺		☺	☺	☺
Gong 2016 [48]	☺	?	?	☺		☺	☺	☺
Winklhofer 2016 [63]	☺	?	?	☺		☺	☺	☺
Al-Najami 2017 [50]	?	?	☺	☺		☺	☺	☺
Chuang-bo 2017 [49]	☺	☺	☺	?		☺	☺	☺
Fan 2017 [53]	☺	☺	?	☺		☺	☺	☺
Liang 2017 [37]	☺	☺	☺	☺		☺	☺	☺
Chen 2017 [38]	☺	☺	☺	☺		☺	☺	☺
Liu 2017 [46]	?	☺	?	☺		☺	☺	☺
Meng 2017 [47]	☺	?	☹	☹		☺	☺	☺
Yang 2018 [42]	☺	?	?	☺		☺	☺	☺
Xie 2018 [41]	☺	☺	?	☺		☺	☺	☺
Sun 2018 [52]	☺	☺	?	☺		☺	☺	☺
Lourenco 2018 [62]	☺	☺	?	☺		☺	☺	☺
Li 2018 [39]	☺	?	?	☺		☺	☺	☺
Kang 2018 [54]	☺	☹	☹	☺		☺	☺	☺
Kim 2018 [56]	☺	☺	☺	☹		☺	☺	☺
Ge 2018 [59]	☺	?	?	☺		☺	☺	☺
Cheng 2018 [44]	☺	☹	?	☺		☺	☺	☺
Zhang 2019 [32]	☺	?	?	☺		☺	☺	☺
Zhou 2019 [31]	☺	☺	?	☺		☺	☺	☺
Yang 2019 [60]	?	?	?	☺		☺	☺	☺
Wu 2019 [55]	☺	☺	☹	☺		☺	☺	?
Kupelli 2019 [43]	☺	☺	?	?		☺	☺	☺
DeKock 2019 [58]	☹	?	☺	☹		☺	☺	☺
Al-Najami 2019 [51]	?	☺	☺	☺		☺	☺	☺
Dane 2020 [57]	☺	☺	?	☺		☺	☺	☺

Figure S1. Risk of bias and application concern assessment using the QUADAS-2 tool. Studies are listed chronologically based on publication year. Symbols defining the following: low (☺), high (☹), or unclear (?).
